# Iranian university students lifestyle and health status survey: study profile

**DOI:** 10.1186/s40200-017-0329-z

**Published:** 2017-12-20

**Authors:** Masoume Mansouri, Farshad Sharifi, Mehdi Varmaghani, Hamid Yaghubi, Yousef Moghadas Tabrizi, Maede Raznahan, Alireza Khajavi, Maryam Ghodsi, Payam Roshanfekr, Gita Shafiee, Abasali Keshtkar, Mahdi Ebrahimi

**Affiliations:** 1 Student Health Services, Students’ Health and Consultation Center, Tarbiat Modares University, Tehran, Iran; 2 Elderly Health Research Center, Endocrinology and Metabolism Research Institute, Tehran University, Tehran, Iran; 3 Social Determinants of Health Research Center, Mashhad University of Medical Sciences, Mashhad, Iran; 4 Department of Psychology, Shahed University, Tehran, Iran; 5 Department of health and sport medicine, faculty of physical education and sport science, University of Tehran, Tehran, Iran; 6 Noor Ophthalmology Research Center, Tehran, Iran; 7 Department of Epidemiology and biostatistics, Tehran University of Medical Sciences, Tehran, Iran; 8 Faculty of Paramedical Sciences, Shahid Beheshti University of Medical Sciences, Tehran, Iran; 9 Non-communicable Diseases Research Center, Endocrinology and Metabolism Population Sciences Institute, Tehran University of Medical Sciences, Tehran, Iran; 10 Diabetes Research Center (DRC), Endocrinology and Metabolism Research Institute (EMRI), Tehran University of Medical Sciences (TUMS), Tehran, Iran; 11 Social Determinant of Health (SDH) Research Center, University of Social Welfare and Rehabilitation Science, Tehran, Iran; 12 Chronic Diseases Research Center, Endocrinology and Metabolism Population Sciences Institute, Tehran University of Medical Sciences, Tehran, Iran; 13 Department of Health Sciences Education Development, School of Public Health, Tehran University of Medical Sciences, Tehran, Iran; 14 Department of Internal Medicine, Sina hospital, Tehran University of Medical Sciences, Tehran, Iran

**Keywords:** Risky health behaviors, Physical health, Behavioral patterns, University students

## Abstract

**Background:**

The physical health assessment of university students in Iran is a national large scale assessment examining health behaviors among tertiary education students. Understanding risky health behaviors which are the major sources of global mortality and morbidity in adulthood is the key objective of this assessment.

**Methods:**

In academic year of 2012–2013, newly admitted students (*N* = 151,671) at 74 governmental eligible universities that had health center from 28 provinces were invited to participate in the health assessment program. The physical health behaviors of the students were evaluated by using questionnaire. The test-retest reliability method was applied to estimate the reliability of physical health questionnaire. After filling out the questionnaires*,* students were led to the examination room for the measurement of height, weight and blood pressure.

**Results:**

From the total study population, 84,298 student’s ages between 18 and 29 years old, were participated in the health assessment. The mean response proportion was 63%. The mean age of students was 21.5 ± 4.01, with 49.20% percent being <20 years old. 32.31% were between 20 and 24 years, 13.44% between 25 and 29 years, 69% of the participants were undergraduate 34.9% were master’s students, and 2.9% were Ph.D. students. The mean BMI for total students was 22.5 ± 4.0 and regarding to gender, the mean BMI for male and female were 23.0 ± 4.1 and 22.2 ± 3.8 respectively.

**Conclusion:**

Analysis of student’s findings will generate multiple studies which report different aspects of physical health of Iranian university students who constitute a large proportion of young adult aged 18–29 years in the country. This assessment also provides opportunity to compare Iranian student’s behavioral patterns with the behavioral pattern of students worldwide.

## Background

### The importance of evaluating health behavior in young population

Risky health behaviors are the major sources of global mortality and morbidity in adulthood and the five leading causes of death at a young age are rooted in health risky behaviors [1–3]. For these, development of a youth study program to address risky health behaviors was strongly recommended [4]. Studies also showed, behavioral habits in young people have a direct impact on the risk of developing non-communicable disease (NCDs) later in life [5, 6]. In this, NCDs already account for over 70% of the mortality observed in Iran [7]. Given that health problem in adulthood are preventable by influencing youth behavior [1], health professionals are interested in identifying and correcting the behaviors that causes NCDs at a young age [5].

Iran is home to 24 million young people between the 15–29 years and the health of this population is a priority. According to last census**,** 25% of total Iranian youth population study at universities [8, 9].Considering tertiary level education, UNESCO educational attainment dataset reveals that, among the countries in the Middle East region, Iran has the highest number of educated people at bachelors and master’s degrees level [10]. In this regard one of the best way to assess the effect of risk factors on the prevalence of common disease in various societies, is through population cohort studies [11]. In the majority of the world’s population cohort studies, the youngest age group enrolled, was 35 years and above. [12–16] which is attributed to lack of access to age groups of 18 to 30 years at the community level or high rates of attrition. Therefore, in countries with high admission rates in academic education, health assessment of university students seems to be an alternative option to obtain health information in these age groups.

Furthermore, the number of studies evaluating health assessment in the college students at the international level is limited in various countries. The only major assessment in the world is the Survey entitled “American College Health Association-National College Health Assessment (ACHA-NCHA)” which was developed by ACHA in 1998 [17, 18]. Similarly, very limited number of studies in Iran have investigated health status of college students with a sample size between 200 and 500 [19–21]. It seems, in this age group there is an information gap in the field of behavioral habits which could be a risk factor for NCDs later in life. It should be noted that this study can have two important reasons for this issue: First, age groups of 18 to 29 years of age, usually in most national health surveys or large-scale studies, are not present. Secondly, major national health surveys in most countries of the world are household or family-based and in countries with a significant student population, such as Iran, these groups will not be included in the surveys. Therefore, it is important to focus on the risky behavior of university students to study NCDs. In this survey, we investigated health related outcomes in terms of health habit and behavior, physical and eating habits and personal and familial medical history, using tobacco and physical health; weight, nutrition, exercise and personal safety.

### Study design

Counseling and health organization of the Ministry of Science and Technology (CHOMST), has designed mental and physical health assessment of university student in Iran: (MEPHASOUS-Iran**)** and wants to implement it in universities health centers. The key objective of this assessment is to understand the present health issues and behaviors among university students.

Among national studies, this is the first large-scale Iranian health assessment study to compare the protective and risky behavior in university students. Based on the need and resources of the university, the staff members of health center were different from a multidisciplinary team, to a physician, a nurse and one psychologist. Today, health center are established in most universities of Iran. Nevertheless, some universities don’t have health center.

### Higher education admission system

The students of governmental universities selected through the national entrance examination which is uniformly designed by the Ministry of Science and Technology (MST). According to the training facilities of the provinces a fixed admission quota system has been set by MST. Under quota system, university students*’* admissions create opportunity for students to enter tertiary level education from all the provinces with different culture and socio-economic situation. Furthermore, this type of selection can increase diversity of selected students who can be representative of different cultures and communities which influence patterns of behavior and habits. This population survey was designed based on the findings of student’s health assessment for newly admitted university students in 2012–2013 academic years in governmental universities across the country.

## Methods

**Fig. 1:**
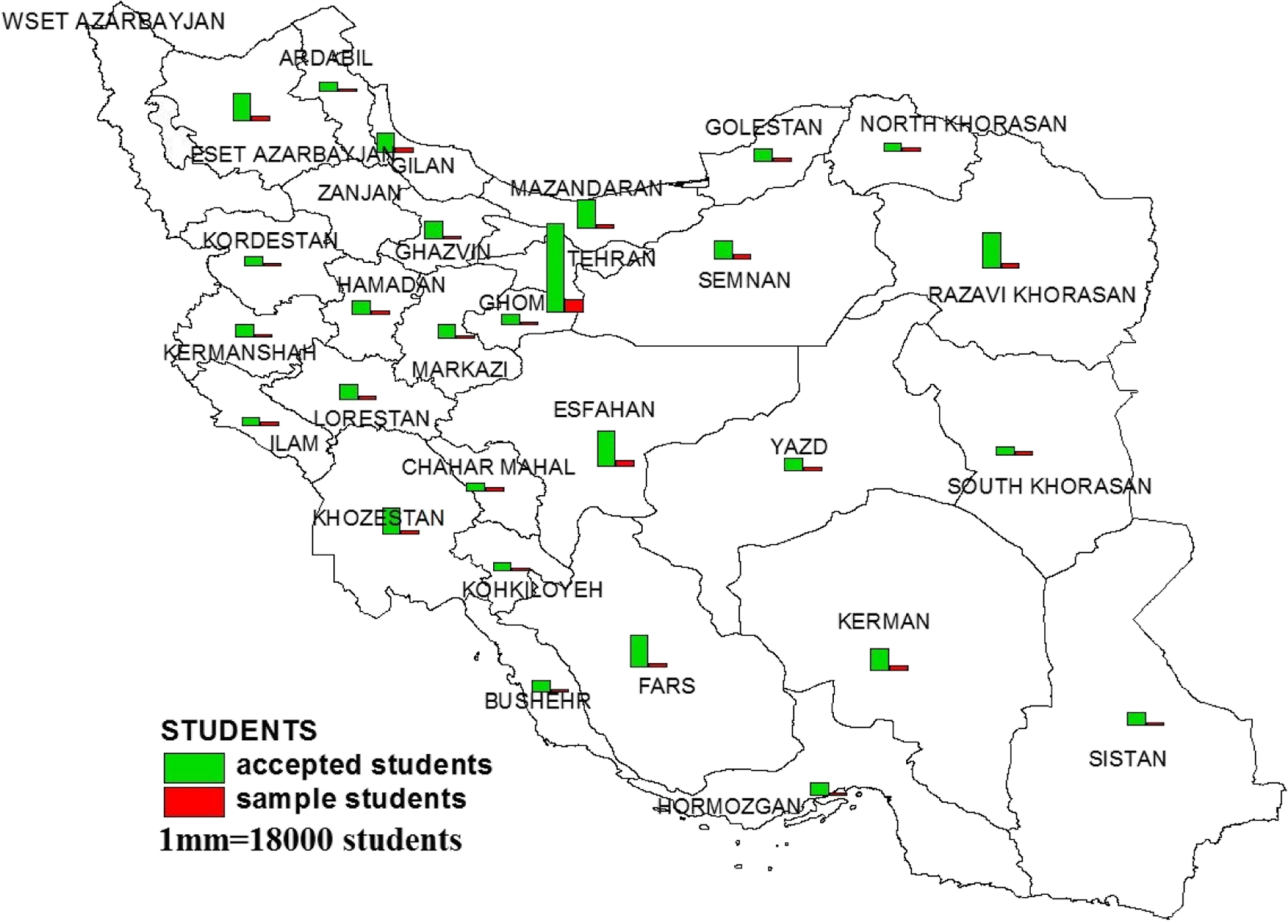
The distribution of accepted and participated students in mental and physical health assessment in university student of Iran (MEPHASOUS-Iran) by provinces (2012–2013 academic year)

**Fig. 2:**
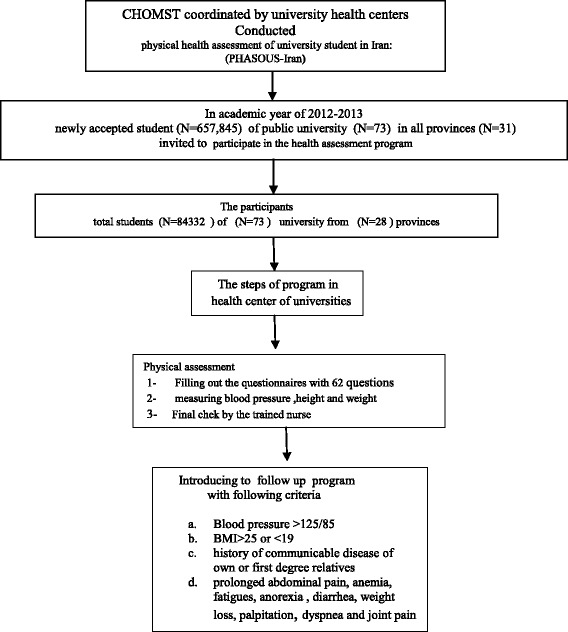
Flow chart of Mental and Physical health Assessment of University Students in Iran

In recent years CHOMST coordinated by university health centers conducted students’ physical health assessment program yearly, so as to evaluate universities health initiatives and map their health data. Instruction of the program was provided in the summer by expert panel of physical health professionals. Self-administered questionnaire was used to conduct lifestyle evaluation. Different parts of the questionnaire were summarized in Table 1.The detail of the program assessment was announced to the health center of the public universities. All public universities from various geographical regions that had health center, participated in the program (Fig. 1). This round of health assessment program was launched in the beginning of educational year of 2012–2013 academic year and it lasted for over a period of three-months: from October to December, that included two stages: an initial screening and follow up. During data collection, the students who met the criteria for follow up were recalled and introduced to higher level of health provision (Fig. 2).

**Table 1:** Different parts of life style questionnaire

Section	Field of assessment	Number of questions	Sample
First section	anthropometric and demographic	17	age, gender, marital status, grade of education, occupation and insurance condition
Second section	personal habits	7	physical activity, smoking, tooth brushing habits, sleep pattern /alcohol consumption, hours working with the computer
Third section	dietary habits	13	daily use of diary product, fruit, vegetables, fast food, junk food
Forth section	medical history	15	personal and familial medical history
Fifth section	exposing to environmental hazards	10	excessive noise, vibration, radiation, chemical and biologic materials

For all rounds of program and data collection, a similar methodology and time schedule was applied. Administration and storage of information gathered from students were handled in partnership with university health and centers and CHOMST.

### Students enrollment process

All newly admitted students at 74 governmental eligible universities that had health center from 28 provinces affiliated to the MST were invited to participate in the health assessment program. In 2012–2013 academic years, about 151,671students were admitted in governmental universities of Iran at three levels which included undergraduate, master and PhD degree [22].

During the registration period the Universities Health Center announced that it will launch a health assessment program. The students received detailed information regarding the health program by the health center medical staff: who had a hospitality space in registration location. Screening appointment dates and times were determined according to the number of students in each faculty. A number of 50 to 70 students were scheduled daily. The students were requested to view details of the program agenda on the website to determine the time of assessment. The students were also notified of the assessment by E-mail with the assessment link on the website, short message system (SMS) and putting up the posters all over the universities. All students received two reminder SMS that informed them of an upcoming health assessment and provided details about the timing program 1 week before the program initiation and 1 day before the specified time for each faculty. At the end of third month, the program was extended for 2 weeks in order to cover students who failed to attend the program on time. Students who had a medical problem received a follow up reminder.

In the first step, at the physical health section, each student was assigned to a unique ID number, which was embedded in the questionnaire. The information was entered into the database with this number. The medical staff assured students that their information would be kept confidential. Subsequently, students were asked to fill out a self-administered questionnaire which had 62 questions in 5 sections (Table 1). The questionnaire included questions that have been widely used to evaluate the behavior of students. The first and required step was signing the consent form attached to questionnaire.

After filling out the questionnaires*,* students were led to the examination room for the measurement of height, weight and blood pressure. Body weight and height was measured using the Seca weighing scales and a permanently wall mounted stadiometer, respectively. Systolic and diastolic blood pressure was measured for participants in a sitting position by Richter mercury sphygmomanometer.

### Data management

After data collection, the responses of students to physical questionnaire were entered into SPSS version 16 (software) by two different data entry operators and saved into two separate data files. Based on CHOMST instructions, common coding system (code and label) for variables of the questionnaires was applied.

When data entry was complete, a copy of the final data was sent to CHMOST through email. Each of the universities was responsible for data analysis and interpretation*.* However, the aggregate results of all the universities were analyzed and released by the expert teams set up by CHOMST*.*

### Quality assurance and quality control (assessing reliability and validity)

A test-retest reliability process was applied in order to estimate the reliability of health assessment questionnaires measuring physical health behavior of universities students. A sample of 70 students in each center was voluntarily selected to fill the health assessment questionnaires two times with a 2–3 weeks’ interval. To examine the consistency of the results, the correlation between the answers in the first and second time for each of the 70 students measured. To test the accuracy and reliability of measurements, two trained nurses were asked to take systolic and diastolic blood pressure, weight and height for each student during test, retest process with the same instruments.

Therefore 70 students have two information for each question and four recordings for systolic and diastolic blood pressure, weight, height which were taken by two nurses during test and retest independently. Correlation and Kappa analysis were conducted, to examine inter-rater reliability and agreement between two nurses ‘readings [23, 24].

## Results

The participants were from 74 governmental universities located in 28 provinces from the total of 31 provinces, While three of them had no information (Fig. 1). Flow chart of Mental and Physical health Assessment of University Students is summurized in Fig. 2. To overcome the heterogeneity induced by the variation of sex and age group, stratification of the population into homogeneous subgroups including two sexes and 4 age groups were created. From the total population of the study, the data of 84,298 students ages 18–29 years were analyzed (Table 2). Based on the definition of youth in Iran (15-29 years) and to simulate the target population age range, the students above the age of 29 years were excluded from the analysis. Thus the results are presented based on the target population age range*.* In the second step of data cleaning, in order to reduce wide confidence interval of the mean, the data of students under the age of 18 that constitute only 0.3% of students were also excluded.

**Fig. 3:**
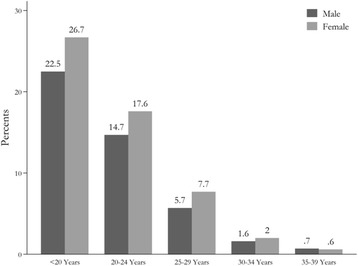
The distribution of participated students by age and sex

**Fig. 4:**
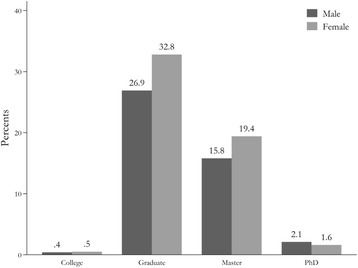
The distribution of participated students by sex and level of education

Combining these two age groups, students between the ages of 18–29 years constituted more than 95% of the student’s population who enrolled in assessment program in 2012–2013 (Fig. 3). The mean age of students was 21.5 ± 4.01, with 49.20% percent being <20 years old. 32.31% were between 20 and 24 years, 13.44% between 25 and 29 years, 3.7% between 30 and 34 years and 1.39% between35–39 years. The Only 4.9% of students were aged over 30 years (Fig. 3).

Table 2 shows the distribution of total students by sex and educational level and various levels of university degree in each province. Sixty-nine percent of the participants were undergraduate, 34.9% were master’s students, while 2.9% were PhD students. In all provinces, most participants were undergraduate and master degree students (Fig. 4).

**Table 2:** The distribution of student sample by sex and level of education in each province

Province	Sex		Level of education						
	Male	Female	College	BS	MA/Ms	MD Field	PhD	other	total
1-Ardabil	253(36.6%)	439(63.4%)	9(1.3%)	558(80.6%)	88(12.7%)	1(0.1%)	31(4.5%)	5(0.7%)	692
2-Boushehr	229(36.2%)	404(63.8%)	22(3.5%)	611(96.5%)	(0%)	(0%)	(0%)	(0%)	633
3-Chaharmahan & Bakhtiyari	698(41.7%)	974(58.3%)	4(0.2%)	1073(64.2%)	538(32.2%)	39(2.3%)	7(0.4%)	11(0.7%)	1672
4-East Azarbayijan	1821(46.9%)	2065(53.1%)	20(0.5%)	2023(52.1%)	1477(38%)	24(0.6%)	316(8.1%)	26(0.7%)	3886
5-Esfahan	2807(46.3%)	3253(53.7%)	37(0.6%)	3330(54.9%)	2360(38.9%)	48(0.8%)	249(4.1%)	36(0.6%)	6060
6-Fars	1128(40.8%)	1634(59.2%)	23(0.8%)	1964(71.1%)	732(26.5%)	1(0.03%)	35(1.3%)	7(0.2%)	2762
7-Gilan	1257(41.8%)	1748(58.2%)	7(0.2%)	2196(73.1%)	713(23.7%)	2(0.1%)	71(2.4%)	16(0.5%)	3005
8-Golestan	429(33.4%)	856(66.6%)	54(4.2%)	879(68.4%)	308(24%)	(0%)	41(3.2%)	3(0.2%)	1285
9-Hamedan	573(40.7%)	836(59.3%)	24(1.7%)	1082(76.8%)	265(18.8%)	10(0.7%)	(0%)	28(2%)	1409
10-Hormozgan	64(15.4%)	351(84.6%)	(0%)	389(93.7%)	24(5.8%)	(0%)	(0%)	2(0.5%)	415
11-Ilam	208(31.5%)	452(68.5%)	29(4.4%)	457(69.2%)	163(24.7%)	(0%)	5(0.8%)	6(0.9%)	660
12-Kerman	2111(43%)	2800(57%)	34(0.7%)	3406(69.4%)	1390(28.3%)	12(0.2%)	62(1.3%)	7(0.2%)	4911
13-Kermanshah	481(40.1%)	718(59.9%)	3(0.3%)	1008(84.1%)	163(13.6%)	10(0.8%)	13(1.1%)	2(0.2%)	1199
14-Khorasan Razavi	2168(40.6%)	3171(59.4%)	43(0.8%)	3524(65.9%)	1505(28.2%)	27(0.5%)	240(4.5%)	(0%)	5339
15-Khouzestan	1430(47.9%)	1553(52.1%)	28(0.9%)	2272(76.2%)	584(19.6%)	27(0.9%)	64(2.1%)	8(0.3%)	2983
16-Kohkillouye & Boyrahmad	203(25.4%)	595(74.6%)	4(0.5%)	571(71.6%)	220(27.6%)	(0%)	(0%)	3(0.4%)	798
17-Kordestan	697(46.7%)	796(53.3%)	13(0.9%)	1088(72.9%)	383(25.6%)	2(0.1%)	7(0.5%)	(0%)	1493
18-Lorestan	343(81.9%)	76(18.1%)	1(0.2%)	335(79.9%)	57(13.6%)	9(2.1%)	17(4.1%)	(0%)	419
19-Markazi	685(44.6%)	852(55.4%)	74(4.8%)	1246(81.1%)	208(13.5%)	0(0%)	8(0.5%)	1(0.1%)	1537
20-Mazandaran	1647(50.7%)	1591(49.1%)	28(0.8%)	1978(61.1%)	1054(32.6%)	4(0.1%)	131(4%)	43(1.3%)	3238
21-North Khorasan	396(55.2%)	321(44.8%)	18(2.5%)	648(90.4%)	51(7.1%)	(0%)	(0%)	(0%)	717
22-Qazvin	357(36.7%)	616(63.3%)	1(0.1%)	657(67.5%)	286(29.4%)	1(0.1%)	28(2.9%)	(0%)	973
23-Qom	1079(61.8%)	667(38.2%)	(0%)	1271(72.8%)	436(25%)	2(0.1%)	37(2.1%)	(0%)	1746
24-Semnan	2130(46.7%)	2432(53.3%)	76(1.7%)	3108(68.1%)	1224(26.8%)	30(0.7%)	97(2.1%)	27(0.6%)	4562
25-South Khorasan	781(44.8%)	963(55.2%)	9(0.5%)	1212(69.5%)	478(27.5%)	1(0.1%)	37(2.1%)	5(0.3%)	1744
26-Systan & Balouchestan	575(45.6%)	687(54.4%)	37(2.9%)	827(65.5%)	316(25%)	38(3%)	33(2.6%)	11(0.9%)	1262
27-Tehran	12,175(49.3%)	12,529(50.7%)	97(0.4%)	10,060(40.7%)	12,743(51.6%)	129(0.5%)	1531(6.2%)	144(0.6%)	24,704
28-Yazd	1663(42.9%)	2212(57.1%)	50(1.3%)	2349(60.6%)	1422(36.7%)	7(0.2%)	46(1.2%)	1(0.1%)	3875
Total	38,187(45.3%)	46,111(54.6%)	759(0.9%)	50,326(59.7%)	29,251(34.7%)	421(0.5%)	3119(3.7%)	422(0.5%)	84,298 (100%)

*BA* A Bachelor of Arts (BA, B.A., AB) is a bachelor’s degree awarded for an undergraduate course or program in either the liberal arts, the sciences, or both

*MA* A Master of Arts (M.A., MA; also Latin: Artium Magister, abbreviated A.M., or AM) is a type of master’s degree awarded by universities in many countries. The degree is usually contrasted with the Master of Science degree

*MD* Doctor of Medicine (MD or DM), it is a first professional graduate degree awarded upon graduation from medical school

*PhD* Doctor of Philosophy, abbreviated as PhD, Ph.D., D.Phil., or DPhil in English-speaking countries and originally as Dr.Philos. or Dr.Phil. (for the Latin philosophiae doctor or doctor philosophiae), is a postgraduate academic degree warded by universities

Table 3 depicts the proportion of students sample to the study population in eligible universities separately. The average response rate of the 74 universities in our sample was approximately 55%. Most of the participants were from Tehran (29.3%). The demographic characteristics of the university students are shown in Table 4.

**Table 3:** The distribution of response rates for universities in different provinces

Number	Provinces	University	Study sample	Study population	Response rate
1	Ardabil	Mohagheghe ardebili	692	3134	0.22
2	Boushehr	Khalij fars	633	1463	0.43
3-	Chaharmahal & Bakhtiyari	Share kord	1672	2207	0.76
4	East Azarbayijan	Tabriz	2115	6310	0.33
		tarbiyat moalem azarbayjan(shahid madani	919	1780	0.52
		Saanati sahand tabriz	852	1016	0.84
5	Esfahan	Esfahan	2537	3415	0.74
		Saanati esfahan	1332	2398	0.55
		Honar esfahan	465	611	0.76
		Kashan	1580	2078	0.76
		Golpayegan	146 .	171	0.85
6	Fars	Shiraz	2092	4393	0.48
		Honar shiraz	92	113	0.81
		Fasa	285	339	0.84
		Salman farsi kazeroon	293	341	0.86
7	Gilan	Gilan	3005	4820	0.62
8	Golestan	Golestan	610	972	0.63
		Gorgan	675	856	0.79
9	Hamedan	Malayer	1409	1502	0.94
10	Hormozgan	Hormozgan	415	1335	0.31
11	Eylam	Eylam	660	2396	0.27
12	Kerman	Tahsilat takmili saanati kerman	146	288	0.51
		Bahonar kerman	2796	3484	0.80
		Saanati sirjan	467	551	0.85
		Jiroft	456	924	0.49
		Valiasr rafsanjan	1046	1754	0.60
13	Kermanshah	Razi	1199	2696	0.44
14	Khorasan Razavi	Ferdosi mashhad	3309	5630	0.59
		Gonbad	576	875	0.66
		Ghoochan	469	713	0.66
		Sabzevar	745	2544	0.29
		Sabzevar fanavari novin	240	295	0,81
15	Khouzestan	Chamran ahvaz	1919	4032	0.48
		Sanat naft abadan	129	261	0.49
		Jondi shapoor dezfool	243 .	509	0.48
		Oloom va fonnon daryaei khoramshahr	327	574	0.57
		Behbahan	365	486	0.75
16	Kohkillouye & Boyrahmad	Yasooj	798	1909 .	0.42
17	Kordestan	Kordestan	1493	2283	0.65
18	Lorestan	Lorestan	419	2450	0.17
19	Markazi	Arak	1208	1439	0.83
		Tafresh	329 .	443	0.74
20-	Mazandaran	Mazandaran	1408	3216	0.44
		Oloom keshavarzi sari	492	794	0.62
		Sanati babol	1338	1362	0.98
21	North Khorasan	Bojnoord	519	611	0.85
		Bojnoord_kosar	15	267	0.06
		Esfarayn	183	254	0.72
22	Qazvin	Beinolmelal emam khomeini	973	1747	0.56
23	Qom	Hazrat masoume	93	97	0.96
		Saanati qom	456	456	1
		Qom	1197	1363	0.88
24	Semnan	Semnan	2457	4829	0.51
		Saanati shahrood	1432	3348	0.43
		Damkhan	673	1029	0.65
25	South Khorasan	Birjand	1747	3626	0.48
26	Systan & Balouchestan	Zabol	987	1500	0.66
		Chabahar	275	332	0.83
27	Tehran	Tehran	7704	15,150	0.51
		Shahid beheshti	3495	3850	0.91
		Shahed	1009	1015	0.99
		Allame tabatabaei	2162	4543	0.47
		Elmo saanat iran	1653	2638	0.63
		Tarbiyat modares	1649		0.63
		Kharazmi	1625	2737	0.59
		Alzahra	1438	3121	0.46
		Saanati sharif	1259	2732	0.46
		Amir kabir	1023	4297	0.24
		Honar tehran	926	926	1
		Saanati khaje nasirodin toosi	658	1722	0.38
		Varamin	103	483	0.21
28	Yazd	Yazd	3462	4441	0.78
		Ardakan	413	423	0.98
	Total		84,298	151,671	0.55

**Table 4:** Description of demographic characteristics of the university students participated in screening program by gender

	Total Participants	Male	Female
Number (%)	84,298 (100%)	38,223(45.3%)	46,075(54.7%)
Age(years)	21.5 ± 4.0	21.5 ± 4.1	21.5 ± 3.9
Weight(kg) (mean ± sd)	64.3 ± 14.1	71.6 ± 14.2	58.3 ± 10.7
Height(m) (mean ± sd)	168.5 ± 9.6	176.3 ± 6.8	162.0 ± 6.1
BMI (mean ± sd)	22.5 ± 4.0	23.0 ± 4.1	22.2 ± 3.8
Blood Pressure systolic(mmHg)	92.4 ± 40.3	99.1 ± 40.0	86.9 ± 39.7

## Discussion

The health assessment is part of screening program which was developed by considering the current health issue of the newly admitted students in universities of big cities that had health center. It was gradually extended to any university around the country that had basic requirements for arranging student health examination including appropriate place and medical staff, as shown in Fig. 1, the data of universities for three provinces were not available.

Regarding the international studies, a variety of surveys like the NCHA, BRFSS, and National Health Interview Survey were used to describe the health behaviors of population groups, as well as college students. The vast majority of these studies were carried out in the USA with few exceptions. Small number of participants were tested in these studies [25]. Nevertheless among these studies, there are two major health assessment which include American College Health Assessment (ACHA) which collected data of 30,263 students from 39 campuses and the Canadian version of this assessment comprised 43,000 students from 41 schools [17, 26]. Therefore, to the best of our knowledge, this is the first and biggest large scale assessment examining health behaviors among tertiary education students at national and even international level with a few exceptions. In the following sections, the benefits of the health assessment will be discussed.

Key strength of this assessment is the repetitive nature of the program design. While the cross-sectional study can inherently be strong, but if a cross-sectional study, especially the large-scale type, occurs every year, or at a given time, can be used to evaluate the time trends of diseases, health outcomes or behaviors or different risk factors. This yearly assessment could map behavioral change among students over a long period. In this regard, regular assessments of college students’ health behaviors have been recommended [27]. Furthermore, the assessment results help to understand whether student’s health behavior and performance can be affected by gender, different field of education and university, student’s cultural background and their city of origin. Some investigation revealed that health habits among university students could be affected by gender and field of education [17, 28]. This assessment also provides opportunity to compare Iranian student’s behavioral patterns with the behavioral pattern of students worldwide. If these two are the same, student health professional can develop and implement the same intervention strategy to reach health goals among students within a shorter period of time.

This assessment was also designed to identify students who are experiencing health problems. Therefore, the second strength of the program is the early detection of some disease. During the program, health provider discovered some serious diseases that the students were not aware of. The diagnosis and management of their disease were outlined in the following steps so as to reach higher level of clinical care. This can affect primary and secondary prevention and can also reduce burden of disease.

### Response rate and generalizability

The health center of universities had the capacity to include all the newly admitted students between the ages of 18–29 years in the health assessment. We noticed that gathering more participants would result in a higher response rate and the sample would be more representative of the total number of students admitted. In this regard, a number of announcements were made to encourage a large number of students wider to participate in this program. The health center advert emphasized on free health care benefit of participating in the program and highlighted the provision of free follow up medical care for students who faced any disease.

Similar to international studies such as National College Health Assessment, participation in the assessment was completely voluntary. Table 3 shows the comparison of response rates by each university in different provinces.

Comparing the mean response rate of this assessment (63%) with international studies showed that, the mean response proportion of ACHA-NCHA Spring 2000 -- 28 schools, 16,024 participants was 54%, ACHA-NCHA II fall 2011 and 2015 were 31 and 15% respectively [29]. The overall response rate in Couper study on drug and alcohol usage was less than 41% and was 21 in Asch study. Historical response rates for colleges on alcohol study which was conducted 5 times were between 28% to 69%. The mean response rate for colleges in the 2005 CAS was 28% [30].

Reducing response rate for higher education researchers is a concern and suggestions to enhance survey response rates is an important and valuable subject of higher education research [31]. Many authors believed that response rates approximating 60% for most research should be the goal of researchers [32].

Therefore achieving a high response rate in this assessment was the main strength which led to the application of the findings of the assessment to the entire student population in each university.

We noticed that nonresponse bias did also exist, but studies showed that greater survey participation has only minimal effect on the conclusions of the survey [33].

In comparison to international survey, our findings showed that health center advertising policy was a good and successful experience that increased response rate to more than 50% in most universities. Nevertheless, when students across the country are called for examination, increasing participation rate by close to 100 % will not be easy. To motivate all students to participate in the assessment, incentive policies should be defined by CHOMST in the future. The idea is that the students who did participate in this health program may consider more to their own health than those who did not participate. Thus this can lead to obtaining more students information which has trend toward more positive view and it influences health student’s decision makers.

Comparison of these findings with a nationally representative database such as the National published data of NCDs [7] could help in understanding whether these findings on student behavior can reflect and present the youth behavior at this age in the society and can be generalizable to the overall population of young age. It is worth noting here, that this profile will be used potentially as a basis for the methodology of studies originated from the health assessment findings. Analysis of student’s findings will generate multiple studies which report different aspects of physical health of Iranian university students.

## Conclusion

The mental and physical mental health assessment of university student in Iran (MPHASOUS-Iran) is a national large scale assessment examining health behaviors among tertiary education students. The results of the assessment will help to gain insight into the frequency of healthy and risky behavior among university students who constitute a large proportion of young adult aged 18–29 years in the country. Due to achieving high response rate in most universities, the results of this assessment are generalizable to students’ population. Nevertheless, generalizability of the results of this study to young population should be further investigated in future studies.

## Abbreviations

ACHA-NCHA: American College Health Association-National College Health Assessment
CHOMST: Counseling and health organization of the Ministry of Science and Technology
MST: Ministry of Science and Technology
NCDs: Non-communicable disease
SMS: Short message system
